# A Novel Peptide-MHC Targeted Chimeric Antigen Receptor T Cell Forms a T Cell-like Immune Synapse

**DOI:** 10.3390/biomedicines9121875

**Published:** 2021-12-10

**Authors:** Stacie Shiqi Wang, Kylie Luong, Fiona Margaret Gracey, Shereen Jabar, Brad McColl, Ryan Stanley Cross, Misty Rayna Jenkins

**Affiliations:** 1 The Walter and Eliza Hall Institute of Medical Research, Immunology Division, Parkville, Melbourne, VIC 3052, Australia; wang.st@wehi.edu.au (S.S.W.); luong.k@wehi.edu.au (K.L.); cross.r@wehi.edu.au (R.S.C.); 2 Murdoch Children’s Research Institute, Parkville, Melbourne, VIC 3052, Australia; 3 Myrio Therapeutics, 6-16 Joseph Street, Blackburn North, Melbourne, VIC 3130, Australia; fiona.gracey@myriotx.com (F.M.G.); shereen.jabar@myriotx.com (S.J.); brad.mccoll@myriotx.com (B.M.); 4 Department of Medical Biology, The University of Melbourne, Parkville, Melbourne, VIC 3052, Australia; 5 La Trobe Institute for Molecular Science, La Trobe University, Bundoora, Melbourne, VIC 3083, Australia

**Keywords:** immunotherapy, immune synapse, Chimeric Antigen Receptor (CAR) T cells, T cell cytotoxicity

## Abstract

Chimeric Antigen Receptor (CAR) T cell therapy is a promising form of adoptive cell therapy that re-engineers patient-derived T cells to express a hybrid receptor specific to a tumour-specific antigen of choice. Many well-characterised tumour antigens are intracellular and therefore not accessible to antibodies at the cell surface. Therefore, the ability to target peptide-MHC tumour targets with antibodies is key for wider applicability of CAR T cell therapy in cancer. One way to evaluate the effectiveness and efficiency of ligating tumour target cells is studying the immune synapse. Here we generated a second-generation CAR to targeting the HLA-A*02:01 restricted H3.3K27M epitope, identified as a possible therapeutic target in ~75% of diffuse midline gliomas, used as a model antigen to study the immune synapse. The pMHCI-specific CAR demonstrated specificity, potent activation, cytokine secretion and cytotoxic function. Furthermore, we characterised killing kinetics using live cell imaging as well as CAR synapse confocal imaging. Here we provide evidence of robust CAR targeting of a model peptide-MHC antigen and that, in contrast to protein-specific CARs, these CARs form a TCR-like immune synapse which facilitates TCR-like killing kinetics.

## 1. Introduction

Adoptive T cell therapy strategies have achieved great success as demonstrated by the United States Food and Drug Administration’s (FDA) approval of Kymriah^TM^ (tisagenlecleucel) [1] and Yescarta^TM^ (axicabtagene ciloleucal) [2]. Chimeric Antigen Receptor (CAR) T cell immunotherapy has revolutionised clinical practice; however, the number of known tumour specific cell-surface antigens available to be targeted using conventional antibodies is limited [3]. Understandably, for decades, the global race to identify novel tumour antigens has focused primarily on sequencing primary tumours to identify tumour specific mutations, of which 85% are in intracellular proteins [4]. Therefore, T cell redirection strategies to intracellular targets will further revolutionise the cancer immunotherapy field by enabling broad, tumour-specific targeting using antibodies. Doherty and Zinkernagel’s discovery that peptide presentation by major histocompatibility (MHC) alleles provided the basic mechanism of T cell receptor (TCR) recognition [5] has been used in recent years as an approach in the cancer immunotherapy field by targeting tumour peptide-MHC (pMHC) using TCR-based therapies (comprehensively reviewed by He et al. [6]). The field has adopted strategies to take advantage of the fact that cancer mutations can be presented by MHC at the cell surface and be recognised by T cells as “non-self” neoeitopes. There are currently over 60 clinical trials of TCR-based immunotherapies in clinical trial including those targeting MAGE-A3 [7], MART-1 [8], NY-ESO [9], gp100 for Melanoma and WT-1 for AML [10,11].

Of the over 520 CAR clinical trials ongoing worldwide, only 64 CARs and their unique targets are being utilized [3]. The majority of cancer specific mutations are intracellular proteins, many of which are presented by MHC. In direct contrast to this, the majority of known CAR and TCR targets are tumour-associated proteins [12]. There are known examples of CARs that target HLA-Class I restricted peptides, including NY-ESO-1157-165 [13], EBNA3C, [14] gp100, [15] WT-1 [16], and recently, PHOX2B [17]. Maus, Sadelain and colleagues have reported that CARs targeting intracellular antigens can display enhanced cytotoxic function after engineering to reduce CAR affinity to TCR- like levels [13]. And a recent study demonstrated potent anti-tumour responses by CAR T cells targeting a pMHCI epitope in models with even low MHC expression in models of neuroblastoma [17]. In this study the authors also demonstrate significant interactions of CAR binding to the pMHCI with five out of seven residues of the peptide, as compared to the three or four residues that typically interact with TCR [18]. It is clear that in order to expand the reach and improve the specificity of cancer immunotherapies, targeting neoepitope pMHC complexes using single chain antibodies may widen therapeutic options. The targeting of tumour specific antigens using antibodies that recognise specific pMHCs may have broader applicability in that they can be utilised in various formats including bi-specific antibodies (BiTE), antibody-drug conjugates, as well as many other therapeutic modalities beyond CAR T cells. Therefore, whilst we present CAR T cell data here, there are several alternative approaches that can be employed to re-purpose therapeutic single chain antibodies.

The generation of CAR T cells recognising pMHC class I (pMHCI) is of great interest, due to the capacity of such reagents to target intracellular proteins using both CD8 pMHC class I restricted and CD4 pMHC class II restricted cells. In this study, we characterise the function of a novel single chain antibody CAR targeting the model antigen pMHCI complex H3.3K27M-HLA-A*02:01 and present the first investigation of a CAR T-pMHCI immune synapse. Bioinformatic prediction has predicted binding of the mutant H3.3K27M peptide to HLA*02:01 but not the wild-type peptide [19]. In the course of characterising an antibody against a MHCI restricted epitope, we used a model antigen Diffuse Midline Glioma (DMG) [20,21,22] peptide variant, which resulted in a replacement of lysine by methionine at position 27 (K27M) in 70–80% of DMG. Furthermore, there are few CARs in existence that target pMHC, and therefore there is much to learn about CAR-pMHC interactions. Immune synapses are highly dynamic and involve structural reorganisation at the plasma membrane, with a close interplay of subsequent signalling events allowing T cell function. Understanding the interplay of cytoskeletal elements with intracellular signalling pathways that control cytotoxicity will inform future CAR design.

The novel single chain antibody CAR targeting H3.3K27M-HLA-A*02:01, herein referred to as GCT615-CAR, is a second-generation CAR comprised of a short chain variable fragment (scFv) antibody binding the peptide-MHC target, a hinge-linker region, a CD28 transmembrane domain, a CD28 co-stimulatory signalling domain, and an intracellular CD3ζ signaling tail. A CAR T cell approach offers different therapeutic opportunities, compared to peptide vaccination or a TCR-based approach. Being independent of CD4 or CD8 for activity when binding to target cells, peptide-MHC directed CARs can function in both CD4^+^ and CD8^+^ T cells [23].

While there are previous publications showing the feasibility of peptide-MHC CARs, Refs. [13,24] there remains a significant lack of understanding surrounding the biology of such CARs. How these receptors differ from TCR when engaging with target cells, and specifically what kind of immunological synapse (IS) is formed with cognate antigen and the kinetics of killing, has not been previously described. Further, the role of affinity and avidity when designing peptide-MHC targeted CARs is still not well understood [13]. Previous studies have shown that the TCR-mediated and CAR-mediated immune synapses differ [25], and the field is yet to fully understand how these different interactions at the immune synapse influence T cell function. Engagement of TCR with cognate pMHC on target cells leads to the dramatic reorganisation of cytoskeletal structures forming a well-defined and structured IS. The IS a highly organised cytoskeletal structure that provides stable contact between the T cell and target cell, facilitating the clustering of receptors important for T cell signalling and mediating the directional delivery of cytotoxic granules [26,27].

In a physiological TCR-mediated IS, TCR-pMHC complexes and Lck cluster at the centre of the IS. Within minutes after contact, the microtubule organizing centre (MTOC) polarises and docks at the plasma membrane where TCR signalling has occurred [28]. Concurrently, filamentous (F)-actin is quickly depleted from the centre of the synapse and accumulates at the periphery of the synapse instead [29]. Lytic granules move along microtubules towards the polarized MTOC for coordinated and directional release of cytotoxic granules at the actin-depleted region of the IS [28]. To further enhance perforin function at the target cell membrane, T cells have been reported to use mechanical force during target cell killing, a term coined as mechanopotentiation [30]. This mechanical force is generated by changes to cellular adhesive and cytoskeletal machinery as T cells form synaptic protrusions to increase contact surface area with the target cell. We have previously reported that immune synapses driven by CAR targeting of native cell surface expressed proteins, lack defined “classical” structure, with irregular Lck clustering [25] and incomplete depletion of F-actin from the centre of the IS, as compared to TCR-mediated synapses, possibly demonstrating a more transient interaction with target cells [25,31]. Therefore, T cell cytotoxicity requires a balance of physical and physiological effector functions and how these are influenced by CAR versus TCR targeting is still not yet well understood.

In this study we describe the generation of a novel H3.3K27M-HLA-A*02:01-specific second-generation CAR and investigate its phenotypic and functional immune synapse formation and subsequent effector functions.

## 2. Materials and Methods

### 2.1. Cell Culture

T2 cell lines were a kind gift from Professor Andrew Brooks (Peter Doherty Institute for Infection and Immunity), verified as Mycoplasma negative by the Victorian Infectious Diseases References Laboratory (Melbourne, VIC, Australia) by PCR analysis, and cell line authenticated by Cell Bank Australia. Cells were cultured in RPMI-1640 (Gibco, Life Technologies, Waltham, MA, USA) supplemented with 10% Foetal Calf Serum (FCS, Sigma, St. Louis, MO, USA), 1 mmol/L sodium pyruvate, 2 mmol/L glutamine, 0.1 mmol/L nonessential amino acids, 100 U/mL penicillin–streptomycin, and 20 mmol/L Hepes (all sourced from Gibco). HEK293T cells were obtained from within the Walter and Eliza Hall Institute (WEHI, Melbourne, VIC, Australia) and cultured in DMEM supplemented with 10% FCS (Sigma, St. Louis, MO, USA), 2 mmol/L glutamine (Gibco, Waltham, MA, USA), and 100 U/mL penicillin–streptomycin (Gibco, Waltham, MA, USA). Cells were maintained at 37 °C, 5% CO_2_.

T cells were cultured daily at 0.5 × 10^6^ cells/mL in T cell media, RPMI-1640 (Gibco, Waltham, MA, USA) supplemented with 10% FCS (Gibco, Waltham, MA, USA), 1 mmol/L sodium pyruvate (Gibco, Waltham, MA, USA), 2 mmol/L glutamine (Gibco, Waltham, MA, USA), 0.1 mmol/L nonessential amino acids (Gibco, Waltham, MA, USA), 50 μM β-metamecaptoethanol (Sigma, St. Louis, MO, USA) and rhIL-2 (Peprotech, Rocky Hill #200-02) at 100 IU/mL.

H3.3K27M26-35(RMSAPSTGGV) or negative control gp100(IMDQVPFSV) peptide were obtained from Mimotopes and fresh peptide stocks were used for each experiment. For peptide loading, T2 and HEK293T cells were incubated overnight with 100 μM peptide at 26 °C, 5% CO_2_ [32].

### 2.2. Mice

All mice used were 6–8-week-old wildtype female C57Bl/6 mice, sourced from the WEHI animal facility (Kew, VIC, Australia). They were bred and maintained under WEHI approved, SPF [specific pathogen free] conditions in the WEHI animal facilities (Parkville, VIC, Australia). All experiments were performed under the approval of the WEHI Animal Ethics Committee.

### 2.3. Retained Display Library Panning

The Ruby [1] scFv library Retained Display (ReD) scFv library was constructed using fully-germline IGLV3-1 and IGLV6-57 scaffolds paired with the IGHV3-23 scaffold, as described previously [33].

The Ruby library was panned for two rounds of H3.3K27M-HLA-A*02:01 pMHC complex bound to MyOne Strepdavidin C1 Dynabeads (ThermoFisher Scientific, Vilinius, Cat: 65002). Panned library output was transferred into the ReD cell-display modality [33] and cells were permeabilised with 0.5% n-octyl β-d-thioglucopyranoside (Anatrace, Maumee, Cat: 0314) and labelled using recombinant H3.3K27M-HLA-A*02:01 pMHC labelled with fluorescent dyes excitable at 405 nm and 488 nm (Dy405 and ATTO-488 respectively). Cells that were positive for target binding were isolated using the FACSMelody sorter (Becton–Dickinson, Franklin Lakes, NJ, USA).

After two rounds of positive selection for binding to H3.3K27M-HLA-A*02:01 pMHC complex two further FACS rounds were conducted using counter-labelled HLA-A*02:01 complexes with unrelated peptides. Following four rounds of FACS individual colonies were isolated and grown in 96-well plates before scFv induction, cell permeabilization, and H3.3K27M-HLA-A*02:01 complex labelling and detection by CytoFLEX (Beckman Coulter, Nyon, Switzerland).

Clones that displaying specific binding to the H3.3K27M-HLA-A*02:01 pMHC complex were sequenced and unique scFvs were expressed as N-terminal His6 tag and a C-terminal AviTag [34] fusions (to respectively purify and biotinylate the soluble protein) to the in *E. coli*. Biotinylated scFv protein was released via permeablisation with 0.5% n-octyl β-d-thioglucopyranoside and purified to ~90% purity on Nickel NTA agarose resin (ABT, Madrid, Cat: 6BCL-NTANi).

### 2.4. Alanine Scan and Specificity Analysis of GCT615 scFv

A bead-binding assay was used to quantitate the binding of scFv to HLA-A*02:01 complexes containing either alanine-substituted variants of the K27M peptide, or a panel of 95 complexes featuring unrelated peptide epitopes previously reported to be presented by HLA-A*02:01. Briefly, Streptavidin C1 Dynabeads (ThermoFisher, Cat. No. 65001) were incubated with excess biotinylated scFv before being blocked with free biotin and washed in PBS. Fluorophore-labelled HLA-A*02:01 complex was added to scFV coated beads at a concentration of 3.5 nM and incubated for 1 h at 4 °C followed by 10 min at 25 °C. Binding of the free HLA complex to the beads was quantitated by the CytoFLEX cytometer (Beckman Coulter) at 488 nm (ex)/525 nm (em). Binding was normalized to beads without scFv and with unrelated control MHC complex.

### 2.5. Generation of CAR T Cells

Briefly, HEK293T cells were transfected using FuGENE-6 kit (Catalogue Number E269A, Promega, Madison, WI, USA) with gag/pol packaging vector, ECO mouse trophic envelope vector and transfer vector encoding the second generation CD28z CAR cDNA. T cells were isolated from WT C57Bl/6 mouse lymph nodes by positive selection using either EasySep Mouse positive CD8^+^ T cell isolation kit (StemCell Technologies, Vancouver, BC, Canada) or EasySep Mouse positive CD4^+^ T cell isolation kit (StemCell Technologies, Vancouver, BC, Canada). T cells were then activated using aCD3/CD28 Dynabead (Invitrogen, Calsbad) and transduced with retrovirus on Retronectin (Takara Bio, Shiga, Japan) coated plates and incubated at 37 °C, 5% CO_2_. Cells were generally assayed 7–10 days after transduction, and adjusted for MYC/mCherry transduction efficiencies, for functional assessment.

### 2.6. Flow Cytometry

Cell surface CAR expression was assessed using flow cytometry by labelling with anti-MYC antibody (Clone 9B11, Cell Signaling, Danvers, MA, USA) at 4 °C for 45 min and detection of intracellular mCherry expression. Confirmation of the peptide-MHC complex on the cell surface of target cells were performed with staining on ice for one hour, with a recombinant scFv tetramer-Atto-488. 

For analysis of T cell activation, CAR T cells and tumour cells were co-incubated for 10 h in T cell media at a 1:1 ratio at 37 °C and 5% CO_2_, in triplicate. Positive control for CAR stimulation was provided using plate bound unconjugated MYC-tag (Clone 9B11, Cell Signaling, Danvers, MA, USA), pre-coated overnight 4 °C at in PBS. Cells were then incubated with anti-CD25 APC (Clone PC61, BioLegend, San Diego, CA, USA), anti-CD69 PerCP (Clone H1.2F3, BD Biosciences, East Rutherford, NJ, USA), anti-CD4 FITC (Clone GK1.5, produced at WEHI Bundoora), and anti-CD8 PE antibodies (Clone 53-6.7, BD Pharmingen, East Rutherford, NJ, USA) at 4 °C for 30 min. Cells were analysed on a Fortessa X20 (BD Biosciences, East Rutherford, NJ, USA) and analysis performed on FlowJo software (TreeStar, Ashland, OR, USA). For analysis, cells were gated on live cells, CD4 and CD8, then CD25 and CD69.

### 2.7. Chromium Release Assay

Cytotoxicity was examined by labelling the target cells with 100 mCi 51Cr for 1 h at 37 °C in T cell media, before excess 51Cr was washed off and target cells cultured at 1 × 104/well in a 96 well plate. CAR T cells were then added at varying effector:target (E:T) ratios. The plate was incubated at 37 °C, 5% CO_2_ for 4 h and supernatant harvested to a fresh plate. 51Cr levels in supernatant was read using a β-counter (Luminescence Counter, Perkin-Elmer, Waltham, MA, USA) and the percentage of lysis calculated using the following formula: [(experimental counts/minute-spontaneous counts/minute)/(total counts/minute-spontaneous counts/minute)] x 100. The level of 51Cr release from targets alone did not exceed 10% of the total 51Cr release from targets lysed with 1% Triton X-100 to determine maximal lysis, and results are shown as the percentage of cytotoxicity.

### 2.8. Cytokine Bead Array

1 × 10^5^ effector CAR T cells and target cells (in the presence or absence of peptide) were cultured at a 1:1 ratio, or for the positive control, 1 × 10^5^ CAR T cells were plated alone into plates pre-coated with anti-MYC antibody. Cultures were incubated at 37 °C, 5% CO_2_ for 4 h before harvesting 10 mL of culture supernatant and assaying for cytokine and chemokine secretion using CBA flex sets (BD Biosciences, East Rutherford, NJ, USA) according to the manufacturers instructions. We assayed for mouse IFNγ, TNF, IL-2, Mip1a (CCL3), Mip1β (CCL4) and RANTES (CCL5). Samples were analysed on a FACS verse and FCAP array software version 3.0 (BD Biosciences, East Rutherford, NJ, USA) was used to analyse the data.

### 2.9. Intracellular Cytokine Staining

CAR T cells and tumour cells were co-incubated for 5 h in T cell media at a 1:1 ratio in the presence of Golgi-stop (Becton Dickinson, Kit #554715, final concentration 5 ml/mL), at 37 °C, 5% CO_2_. Cells were subsequently incubated with antibodies to surface proteins in FACS buffer (Phosphate Buffered Saline (PBS) with 0.2% Bovine Serum Albumin (BSA, Gibco)), with anti-CD4-BV421 (Clone MP6-XT22, BD Horizon, Franklin Lakes, NJ, USA), anti-CD8-BV711 (Clone 53-6.7, BioLegend, San Diego, CA, USA), and the viability dye Fixable yellow (Invitrogen, Oregon) for 30 min at 4 °C. Cells were then fixed for 20 min at room temperature and permeabilised using BD Pharmingen Fix/Perm kit (Cat: 554715, BD Pharmigen, Franklin Lakes, NJ, USA) according to manufacturer’s instructions. Intracellular IFNγ, IL-2 and TNFα were detected by incubating with anti-IFNγ-APC (Clone XMG1.2, BD Pharmingen, Franklin Lakes, NJ, USA), anti-IL2-PE (Clone JES6-5H4; BD Biosciences, East Rutherford, NJ, USA), and anti-TNFα-AF488 (Clone MP6-XT22; Biolegend) antibodies for 1 h at 4 °C. Cells were washed with a FACS buffer and analysed using a Fortessa X20 (BD Biosciences, East Rutherford, NJ, USA) and analysis performed on FlowJo software V10.8 (TreeStar, Ashland, OR, USA).

### 2.10. Live Cell Microscopy

Interactions between CD8+ K27M-specific CAR T cells and tumor cells were assessed by time-lapse live cell microscopy in a 37 °C and 5% CO_2_-controlled chamber, using a previously published protocol [35]. T cells were labelled with 1 µM Fluo-4-AM+ 0.02% (wt/vol) Pluronic F-127 carrier (ThermoFischer, Waltham, MA, USA) at 37 °C, 5% CO_2_ for 20 min. T cells were then added to Ibidi µ-Slide chambers (Ibidi, Martinsried) containing target cells and 200 mM of propidium iodide (ThermoFischer, Waltham, MA, USA). Chamber slides were mounted on a heated stage within a chamber maintained at 37 °C and constant CO_2_ concentrations (5%) and infused using a gas incubation system with active gas mixer (Ibidi). Optical sections were acquired through the centre of the cells by sequential scans of fluo-4 (excitation 488 nm) and PI (excitation 561 nm) or brightfield on a Zeiss LSM 980 or Zeiss LSM 880 using a 20X (NA 0.80) air objective and ZEN 2.3 software Blue Edition (Zeiss, Stuttgart, Germany). For the 488 and 561 channels, the pinhole was set to 4.2 AU, giving a section thickness of 5 μM and XY pixel size of 378.8 nM. Images were acquired between frames every 10–20 seconds for up to 180 minutes.

### 2.11. Fixed Cell Confocal Immunofluorescence of Immune Synapse

Immunofluorescence was used to visualise the immune synapse between CD4+ and CD8+ CAR T cell-tumour cell conjugates. T cells and tumour cells were co-cultured at 1:1 ratio in serum-free media for 5 min before being adhered onto glass slides for 20 min. Cells were fixed and permeabilised for 5 min with 100% −20 °C methanol on ice and blocked with blocking buffer (PBS + 2% bovine serum albumin (BSA)) for 1 h at room temperature (RT). Slides were incubated with primary and then secondary antibodies in staining buffer (PBS+ 0.2% BSA) in a humidified chamber at RT for 1 h and 30 min respectively. Primary antibodies used include mouse anti-Lck (clone 3A5, Merck-Millipore, Burlington, NJ, USA), polyclonal rabbit anti-Actin (C-terminal) (Clone A2066, Sigma, St. Louis, MO, USA) and rabbit anti-γ Tubulin (clone GTU-88, Merck-Millipore, Burlington, NJ, USA). Secondary antibodies used are goat anti-mouse IgG-AF555 (#ab150118, Abcam, Cambridge, UK) and goat anti-rabbit IgG-AF488 (#ab150081, Abcam, Cambridge, UK). Nuclei were then stained with 1 μg/mL of Hoechst 33,342 (ThermoFischer, Waltham, MA, USA) in PBS and 0.2% BSA for 5 min at RT before mounting with 1.5 coverglass and Mowiol. Samples were examined with a Zeiss confocal LSM 980 or Zeiss LSM 880 with lasers exciting at 405, 488, 561 and 633 nm, with a 100× objective. CD4 group had at least 40 synapses per group across at least 25 fields of view and the CD8 group had at least 96 synapses per group across 22 fields of view. Data was pooled across two biologically independent experiments and presented as pooled data from various fields of view. xz reconstructions were processed with the ZEN 2.3 software (Blue Edition), ImageJ v1.52p [36] and rendering in Imaris v9.6 (Bitplane AG, Concord, MA, USA).

Quantification of the immune synapse was based on a 4-point scoring system (Supplementary Figure S1). The higher the scores indicated increasing topological similarity to a classical TCR-mediated immune synapse (referred to therein as physiological) [37] immune synapses. A score of 3 was given to the clear presence and similarity of such each measured parameter to physiological immune synapses. Conversely, the absence of the measured features were given a score of 0. Each immune synapse is scored out of a possible maximum of 15 points using the below formula:

*Total Score = 2 × (parameter_1_) + 2 × (parameter_2_) + morphology*

### 2.12. Statistical Analysis

Statistical analyses were performed using GraphPad Prism 8 software (GraphPad, San Diego, CA, USA). Statistical tests applied include Student *t*-test and two-way ANOVA with post-hoc analysis using Tukey’s multiple comparisons; specific statistical method used is indicated in figure legends. Asterisks within figures refer to statistical difference between test and control groups, P values and the number of replicate experiments performed to derive the data are indicated in the figure legends.

## 3. Results

### 3.1. Generation of Novel Peptide-MHC CAR

**Figure 1:**
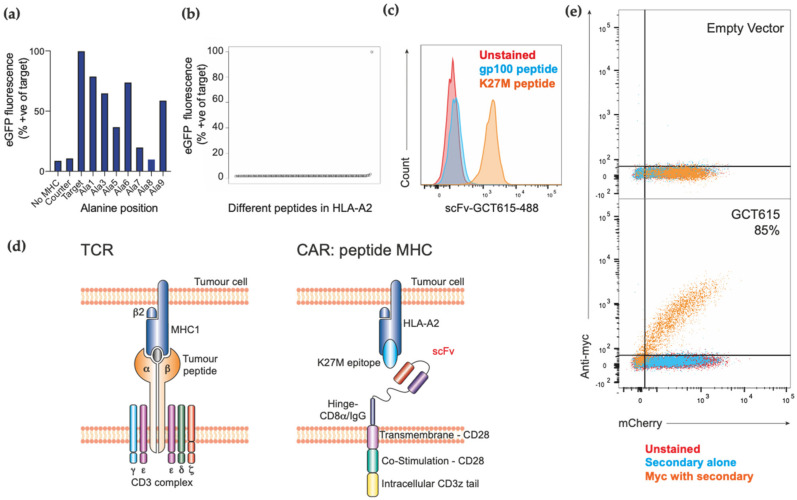
Generation of H3.3-K27M-specific CAR T cells. (**a**) Binding of GCT615 to H3.3K27M-HLA-A*02:01, and to related HLA complexes composed of alanine-substituted peptide epitopes. Demonstrates loss of GCT615 binding when alanine is substituted at position 7 or 8 of the peptide. (**b**) Binding of GCT615 to a native HLA-A*02:01 peptide panel known to be presented at the cell surface (specificity panel). No significant binding to off-target complexes is observed. First data point corresponds to negative control lacking scFv; right-most data point is positive control binding to target H3.3K27M-HLA-A*02:01complex. (**c**) Flow cytometry histogram displaying monomeric GCT615 scFv-488 binding to the HLA-A2.H3.3K27M peptide-MHC complex of T2 cells, either unlabelled (red), or pulsed H3.3-K27M peptide (orange), or the negative control gp100 peptide (blue). (**d**) A schematic diagram of a TCR versus a CAR targeting peptide:MHC complex; (**e**) Flow cytometry labelling of primary murine T cells transduced with either mCherry alone empty vector CAR (top panel), or the GCT615-CAR (bottom panel), showing transduction efficiency of the CAR as determined by mCherry and labelled with anti-myc antibody (Clone 9B11, Cell Signaling, Danvers, MA, USA), with secondary anti-mouse IgG AF488 (goat polyclonal, ab150117, Abcam).

We generated single chain variable fragments antibodies (scFv) against the model antigen H3.3K27M-HLA-A*02:01 peptide-MHC complex by Retained Display screening, with an affinity of 31 nM [33]. To characterise the binding footprint of a scFv clone, designated GCT615, for the H3.3K27M26-35 peptide in HLA-A*02:01, an alanine scan was performed (Figure 1a). A panel of HLA complexes was generated, each carrying a variant of the H3.3 peptide in which a single amino acid residue was substituted for alanine. By measuring binding of the scFv to the variant peptide-HLA complexes, it is possible to examine to contribution to scFv binding made by individual amino acids in the target peptide complex.

It has previously been shown that residue 2 (Lys^®^ Met mutation) serves as an important anchoring residue into the MHC cleft, and therefore the H3.3WT peptide (RKSAPSTGGV) fails to bind due to the extremely low affinity for MHC (H3.3K27M = 151 nM and H3.3WT ≥ 38,687nM) [19]. Furthermore, we found that a loss of scFv binding to peptide after alanine substitution at position 7 or 8 suggests that these positions are important residues for GCT615 scFv recognition. Alanine substitution at positions 3 and 5 of the peptide epitope also resulted in reduced scFv binding to the HLA complex, indicating that the interaction of GCT615 with H3.3K27M-HLA-A*02:01 is highly dependent on the sequence of the peptide moiety. Furthermore, binding of ~70% was maintained with substitution of serine to alanine at position 6 showing that GCT615 is likely to be cross reactive with both H3.3K27M and H3.1K27M (Figure 1a). 

A panel of 95 unrelated HLA-A*02:01 complexes was used to further examine the specificity of GCT615. The specificity panel is composed of fluorescently labelled HLA-A*02:01 complexes containing native peptide epitopes known to be presented by human cells and selected for stability of peptide-HLA complex. Microbeads coated with scFv were incubated with the specificity panel, and binding of the scFv to HLA complexes quantitated by flow cytometry (Figure 1b). No significant binding of GCT615 was observed to any of the HLA-A*02:01 complexes contained in specificity panel, indicating that GCT615 shows excellent specificity for the H3.3K27M-HLA-A*02:01 complex. The absence of off-target binding also indicates that GCT615 has minimal interactions with residues of the HLA-A*02:01 protein scaffold.

Next, we examined the specificity of binding of GCT615 scFv tetramers to HLA-A*02:01 positive T2 target cells, pre-loaded with either H3.3K27M26-35 or gp100 control peptide, both of which are HLA-A*02:01 restricted. Specific GCT615 tetramer binding to H3.3K27M26-35 peptide loaded cells, and not gp100 loaded targets, was detected by flow cytometry, demonstrating specificity of the novel scFv for H3.3K27M antigen (Figure 1c), and confirming the capacity of GCT615 to recognise the target complex when presented by native HLA-A*02.01 at the cell surface. Having demonstrated specificity for a scFv clone (GCT615–see methods), we generated a second-generation retroviral CAR vector comprising of a GCT615 scFv, MYC-tag sequence, CD8a hinge containing a MYC-tag sequence, CD28 transmembrane and co-stimulation domain as well as a CD3x signalling tail IRES mCherry (Figure 1d). Flow cytometry analysis demonstrated excellent transduction efficiency, demonstrated by IRES mCherry expression, and robust CAR cell surface expression (~85%) as determined by MYC-tag staining (Figure 1e).

### 3.2. The H3.3K27M CAR Demonstrates Cytotoxicity against K27M Loaded Cell Lines

**Figure 2:**
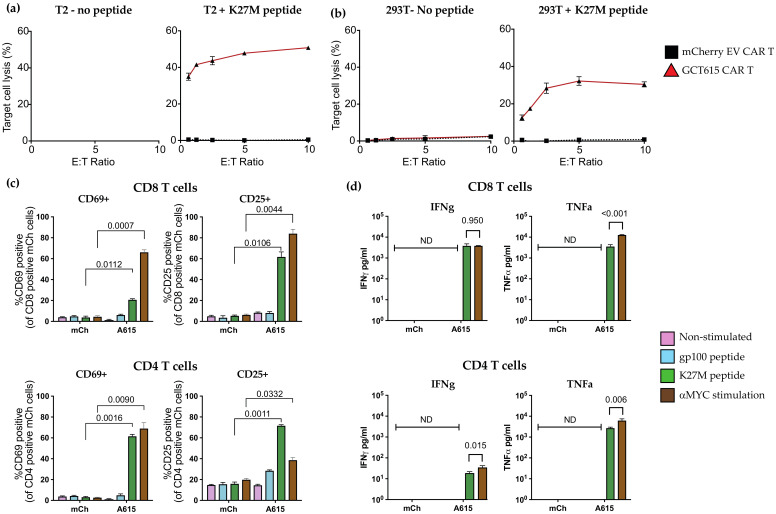
Functional capacity of H3.3-K27M-specific CAR T cells. Cytotoxic capacity of GCT615-CAR T cells as assayed by 4 h co-culture with chromium labelled target cells. CAR T cells were cultured with either: (**a**) T2 cells or (**b**) 293T cells, with or without K27M peptide. Shown is the mean 4 h% target cell lysis in triplicate + STDEV, representative of 4 biological replicates. (**c**) Activation markers CD25 and CD69 expressed on CD4 and CD8 GCT615-CAR T cells, after 24-h co-culture with either T2-K27M, or T2-gp100 targets, or left unstimulated, at a 1:1 effector: target ratio. CAR T cells were activated with anti-MYC antibody as a positive control. Shown is mean% T cells + STDEV of triplicates from one experiment, representative of three biological repeats. Statistical significance was determined by two-way ANOVA with Sidak’s multiple comparisons, *p* values as indicated; ns = not significant. (**d**) Cytokine bead array quantification of supernatants obtained from 24 h co-cultures of either CD4^+^ or CD8^+^ GCT615-CAR T cells and T2 targets at a 1:1 effector: target ratio, or cultured on plate bound anti-MYC antibody, serving as a positive control. Shown is mean% T cells + STDEV of triplicates from one experiment, representative of two biological repeats. Statistical significance was determined by unpaired multiple *T*-test, *p* values as indicated; ns = not significant.

We first examined the ability of the novel GCT615-CAR T cells to specifically induce target cell death and produce cytokine in response to H3.3K27M-HLA-A*02:01. Naïve primary T cells were activated with aCD3/CD28 beads before viral transduction with either the empty vector CAR (mCherry alone) or the GCT615-CAR, and then assessed for effector function 7–10 days after transduction. The GCT615-CAR demonstrated excellent short-term cytotoxicity by chromium release assay against H3.3K27M+ loaded, HLA-A*2:01 T2 target cells (Figure 2a) and good killing of H3.3K27M+ loaded, HLA-A*2:01 293T target cells (Figure 2b), with no background cytotoxicity detected.

Co-cultures of activated CART cells and targets were then analysed for their capacity to express the markers of activation CD25 and CD69. The CAR contains a MYC tag in the stalk, allowing us to non-specifically activate the T cells via the CAR using plate bound anti-MYC antibody as a positive control. MYC-activated GCT615 CAR T cells displayed strong expression of the activation markers CD25 and CD69. Importantly, when the GCT615 CAR was co-incubated with K27M expressing target cells, both CD4^+^ and CD8^+^ T cells demonstrated an excellent ability to activate, displaying elevated levels of CD69 and CD25 expression at the cell surface compared to cells expressing the HLA-A*2:01 -restricted irrelevant peptide gp100 (Figure 2c, blue). As expected, T cells transduced with the empty vector (mCh) did not upregulate CD69 or CD25. 

We next demonstrated that recognition via the GCT615 CAR induces antigen-specific cytokine secretion, by assaying the supernatant of CAR T cells co-cultured with either the gp100-HLA-A*02:01 peptide-MHC complex, or the H3.3K27M-HLA-A*02:01 peptide-MHC complex. The agonistic binding of the protein MYC tag present in the ectodomain of the CAR served as a positive control [38]. Both CD8^+^ and CD4^+^ GCT615 CAR T cells secreted IFNγ and TNFα in response to the HLA-A*02:01/H3.3K27M_26-35_ complex, with no cytokine secretion when co-cultured with cells presenting the gp100 peptide, indicating H3.3K27M-HLA-A*02:01-specific CAR T cell effector function (Figure 2d). The CD4^+^ GCT615 CAR secreted significant TNFα in response to the H3.3K27M-HLA-A*02:01 peptide-MHC complex, demonstrating specific recognition of this peptide-MHC complex, and lower levels but specific secretion of IFNγ.

### 3.3. The H3.3K27M Peptide-MHC CAR Demonstrates Killing by Apoptosis and Pyroptosis

**Figure 3:**
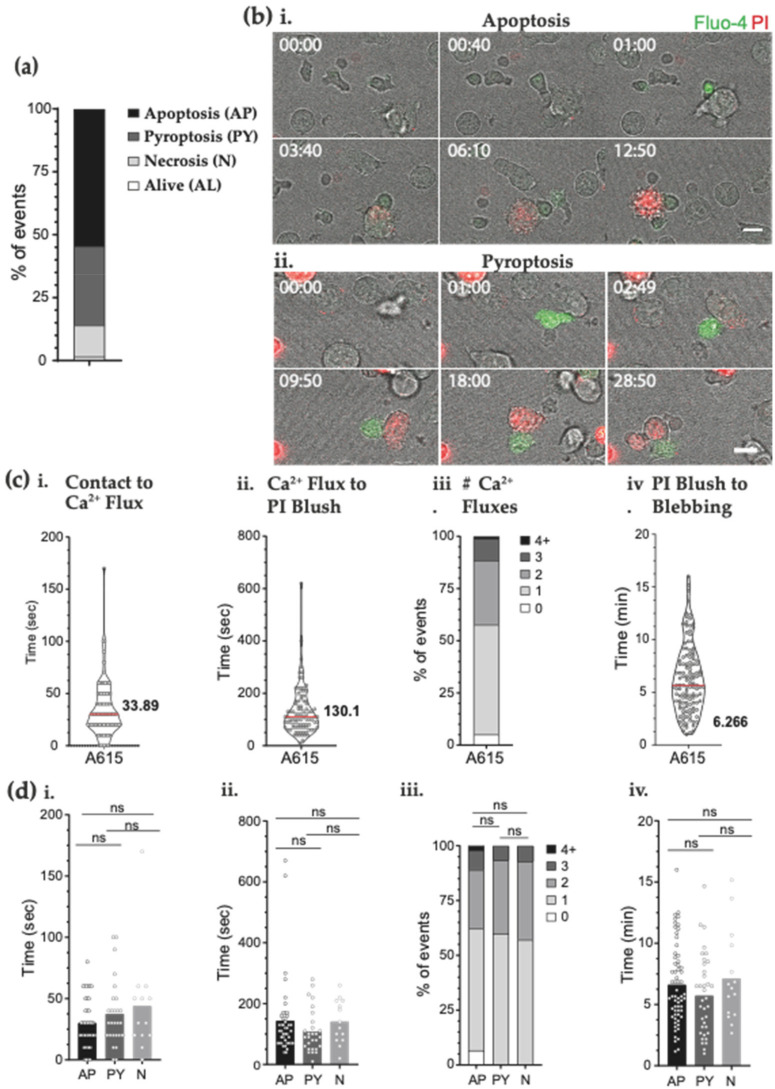
Live cell imaging of H3.3-K27M-specific mouse CD8 CAR T cell cytotoxic response. Time-lapse microscopy of Fluo-4-AM-labelled GCT615-CAR T cells (‘A615′) killing T2-K27M targets in the presence of 200 μM PI. Data pooled from two independent experiments across 26 fields of view. (**a**) Proportion of T2-K27M target cells that had displayed PI uptake after T cell immune synapse formation with a CD8 GCT615-CAR T cell stratified by either apoptosis (AP), pyroptosis (PY) or necroptosis (N) (54.26%, 31.01% and 12.40% respectively). n = 129 events. (**b**) Representative live cell imaging montage of GCT615-CD8^+^ CAR T cell killing T2 targets. CAR T cells are labelled with calcium flux indicator Fluo-4 (green); propidium iodide is in media at 200 μM (red) showing (i) apoptosis or (ii) necroptosis (Videos S1 and S2 respectively). Time scale mm:ss. Scale Bar is 10 µM. (**c**) Live cell imaging data was quantitated: (i) Time from first CAR T contact to T cell Ca^2+^ flux (seconds); (ii) Time from calcium flux to PI blush (lethal hit) (seconds); (iii) number of Ca^2+^ fluxes in each CAR T cell; and (iv) Time from PI blush (lethal hit) to target cell blebbing (minutes). Mean of violin plots as shown. Each horizontal bar shows median value. (**d**) Quantification of all CAR T cell mediated target cell deaths outlined in panel (**c**), pooled and stratified by mode of cell death. Statistical significance was determined using one-way ANOVA with Dunn’s multiple comparisons test, ns = not significant.

The clear T cell activation, cytotoxicity and cytokine production occurring shortly after CAR ligation demonstrated clear specificity. Target cell cytotoxicity is a key feature of immunotherapy, and recent studies have indicated that CAR T cells can induce distinct and alternative forms of target cell death, in addition to apoptosis [39]. We used time lapse live cell microscopy to examine the interaction of the killer T cells in real time and tracked the morphological changes in target cells killed by H3.3K27M-HLA-A*02:01-specific CAR T cells. We found that 98% of all CD8^+^ CAR T cell contacts result in the death of K27M+ expressing target cells (Figure 3a), a majority through morphologically defined apoptosis (Figure 3b(i) and Video S1). In line with a recent study [39], about 25% of target cell death (Figure 3a) displayed morphological features of pyroptosis, characterised by cellular swelling with bubbles instead of smaller apoptotic bodies (Figure 3b(ii) and Video S2). The timing and order of cytotoxic events have been previously characterised [35,40,41], with both T cell receptor and NK cells displaying an intracellular calcium flux ~100 s after membrane contact. Here, the CD8^+^ GCT615-CAR T cells also displayed rapid calcium flux within 33 s, indicative of rapid T cell signalling (Figure 3c(i)). GCT615-CAR T cells delivered a perforin mediated lethal hit to target cells ~130 s after calcium flux, as indicated by PI uptake through pores binding to cytosolic RNA [35] (Figure 3c(ii)). The majority of CAR T cells displayed only one calcium flux before degranulation, with a smaller proportion of T cells displaying several calcium fluxes before pore formation on the target cell (Figure 3c(iii)). The target cells rapidly displayed signs of cell death after T cell killing, taking between 6–9 min to round up (Figure 3c(iv)). This data clearly demonstrates that the novel H3.3K27M-HLA-A*02:01 complex -specific GCT615-CAR T cells specifically recognise target cells and can rapidly induce target cell death in an antigen-dependent manner.

The mode of cell death can have important consequences for a local tumour microenvironment. We examined whether the CAR T cells targeting peptide MHC induced a morphological apoptotic cell death, as defined by characteristic blebbing of the plasma membrane.

When T cell interactions were categorised by target cell death mechanism, there was no significant differences in the T cell killing kinetics between the groups (Figure 3d).

### 3.4. The Novel Peptide-MHC CAR Forms a Classical TCR-Like Immune Synapse

Previous studies have shown that CAR mediated immune synapses show signs of structural differences to those immune synapses mediated by a TCR interaction [25], and the immune synapse structure has been shown to inform the effectiveness of CAR T cells [31]. Here, we explored the influence of a CAR targeting pMHC on driving structural and molecular changes at the immune synapse.

**Figure 4:**
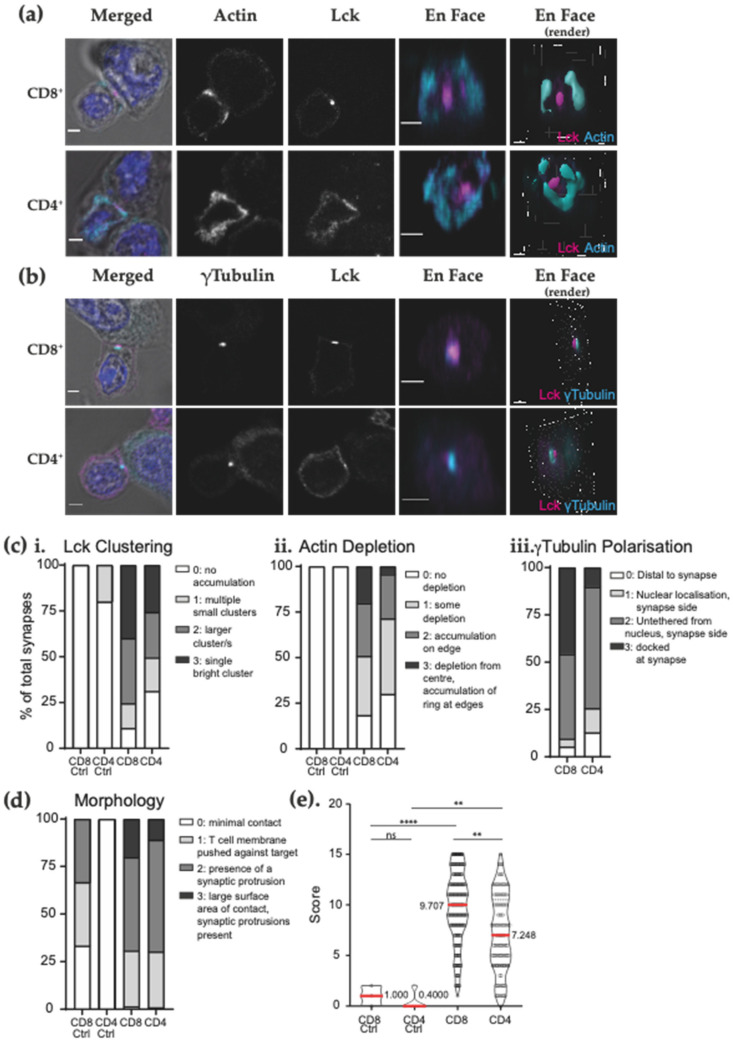
GCT615-CAR T cell target conjugates synapses show topological similarity to physiological immune synapses. CD4^+^ or CD8^+^ GCT615-CAR T cells were co-cultured with T2 target cells before fixing, permeabilising and examining by confocal microscopy and then semi-quantitatively scored as per Supplementary Figure S1. (**a**,**b**) Confocal microscopy of K27M-specific CD4^+^ and CD8^+^ T cells in conjugation with T2 target cells, co-cultured and allowed to adhere to glass slides for 20 min before being fixed, permeabilised and labelled with anti-Lck-488 (magenta) and either (**a**) anti-actin-546 (cyan), or (**b**) anti-γ-tubulin (cyan). Montage of one 2μM plane through the T cell-target pair and ‘en face’ view of the xz plane at the immune synapse. Representative synapses of 2 independent experiments, each with at least 30 synapses. Scale bar, 2 μm. (**c**,**d**). Proportion of GCT615-CAR T cell-T2-K27M immune synapses displaying (**c**) Lck clustering (i), actin clearance (ii) and polarisation of γ-tubulin (iii) (as measured as per Supplementary Figure S1); (**d**) Frequency of T cell morphology of observed engaging immune synapses, as scored on: 0 = minimal contact and 3 = increased surface area, peripheral synaptic protrusions observed (refer to Supplementary Figure S1). (**e**) Pooled total scores of immune synapses in (**a**–**d**), from two independent experiments of at least 30 synapses per experimental group. Detailed description of the scoring system can be found in the methods and see Supplementary Figure S1. Mean of violin plots as indicated. Statistical significance was determined using 2-way ANOVA with Tukey’s multiple comparisons ** *p* < 0.01; **** *p* < 0.0001; ns = not significant. Negative Control synapses (’CD8′ Ctrl and ‘CD4′ Ctrl) created against gp100-pulsed T2 targets whilst experimental synapses (‘CD8′ and ‘CD4′) are K27M-pulsed T2 targets.

Fixed conjugates were examined by confocal microscopy after labelling with antibodies against lymphocyte protein tyrosine kinase, Lck, which marks the central supramolecular activation cluster (cSMAC) [42]. No conjugates (<0.5%) formed in the absence of peptide or when empty vector CAR T cells were examined (data not shown). Using confocal microscopy, 3D reconstruction of K27M-specific CD4^+^ and CD8^+^ CAR T cell IS were semi-quantitatively assessed (Supplementary Figure S1) and demonstrated well-defined clustering of Lck and clearance of F-actin from the centre of the IS (Figure 4a), similar to images previously observed in classical TCR-mediated immune synapses [43]. There was clear clustering of Lck at the immune synapse after engagement of GCT615-CAR T cells with H3.3K27M-HLA-A*02:01. The microtubule organising centre (MTOC), identified by γ-tubulin labelling within the immune synapse, show recruitment adjacent to the centre of the cSMAC of CD8^+^ and CD4^+^ CAR T cells (Figure 4b). Interestingly, CD4^+^ CAR T cell synapses displayed a less γ-tubulin polarised immune synapse structure when compared to CD8^+^ CAR T cells, (Figure 4c), with a lower proportion of conjugates recruiting Lck to a tight cSMAC centre and a “bulls eye” morphology of actin clearance (refer to Supplementary Figure S1). Morphologically, both CD4^+^ and CD8^+^ T cells showed similar patterns of contact with target cells: T cells were observed to be in close proximity and spread across the target cell membrane surface, showing actin-rich synaptic protrusions on one or both cell edges in synapse cross section (Figure 4d, and Supplementary Figure S1).

These data indicate that CAR T cells directed towards peptide-MHC targets are able to effectively kill target cells and display immune synapse structures similar to TCR-mediated synapses. We have functionally validated this novel H3.3K27M-HLA-A*02:01-specific GCT615-CAR, and future studies will focus on determination of its effectiveness for DMG brain tumours both in vitro and in vivo.

## 4. Discussion

Chimeric Antigen Receptors (CARs) are an immune sensor, and when engineered to be expressed by T cells, can override the endogenous specificity of the TCR and redirect them to recognise and kill target cells via binding of the highly specific single-chain variable fragment (scFv). While this immunotherapy approach has shown impressive outcomes in haematological cancers, responses in solid tumours have been disappointing. One limitation of the CAR T cell approach has typically been the reliance on targeting cell surface expressed proteins, carbohydrates and lipids [44]. Given that a high proportion of clinically relevant, “high priority” tumour antigens are intracellular proteins (as ranked by a panel from the National Cancer Institute [45]), the capacity to target intracellular antigens with tuneable CAR T cell approaches would greatly increase the therapeutic applicability of this field. The rational design of CARs with tuneable affinities towards pMHC antigens would provide promising immunotherapy alternatives.

In this study, we have generated and characterised a novel CAR that specifically recognises the HLA-A*02:01-restricted H3.3K27M mutant peptide. We demonstrate excellent antigen specificity of K27M-specific CAR T cells, and expression of T cell activation markers and secretion of IFNγ and TNFα in response to the H3.3K27M-HLA-A*02:01 complex. Current CAR approaches have traditionally involved repurposing well characterised monoclonal antibodies into single chain antibodies, fused with T cell receptor mediated signalling machinery. The affinity of antibodies is typically orders of magnitude above TCR mediated interactions, but despite this, Huppa and colleagues have reported CAR T sensitivity to antigens can be 1000-times reduced compared to TCR mediated interactions [46]. TCRs have been shown to have the capacity to respond to a single pMHC antigen, yet they do require cross-linking for stimulation [47].

Previous studies have shown that the functional avidity of the TCR-tumour immune synapse can influence the synapse dwell time for a T cell in contact with a tumour cell–higher avidity interactions induce a high-quality cytokine profile, while lower avidity interactions are more advantageous for rapid cytotoxicity [48]. In this study, the GCT615-CAR T cells displayed good cytotoxicity against K27M^+ve^ target cells, indicating the pMHCI is targetable by CAR T cells. Patients treated on several CAR T cell clinical trials have exhibited antigen escape in response to therapy [49,50] with the most well-studied example being patients who have previously received anti-CD19 CAR T cell therapy relapsing with CD19 negative tumours [51,52,53]. Therefore, deepening our understanding on the CAR design required to instigate T cell signalling and cytotoxicity in response to very few target antigens is of great interest in the field. Furthermore, rapid target cell death has been shown to be essential for T cell detachment from a dying cell, allowing the effector cell to serially kill multiple targets [41]. How TCR or CAR mediated interactions influence this process is yet to be fully explored. 

Understanding the balance between immune synapse quality and functional avidity will be important for the field in rational CAR design. The role of the immune synapse in cytotoxicity has been well established [27,28], with evidence for the important role of the IS in dictating functional outcomes of cells, and the role of examining recruitment of proximal signalling molecules like Lck to the plasma membrane in CAR T therapy [31]. CD3z is located within the CAR cytoplasmic tail, providing the essential signalling required for ITAM phosphorylation, and recruitment of Lck to CD3 is one of the early events to be detected after CAR ligation.

The construct used in our study comprises a CD28z endodomain signalling tail. In our study we show Lck recruitment to the peripheral SMAC (pSMAC) of the IS after CAR ligation, total clearance of actin to the distal SMAC (dSMAC) and polarisation of the MTOC, in both CD4^+^ and CD8^+^ T cells, as shown in Figure 4. CD4 and CD8 coreceptor interactions have been shown to bind MHC and stabilise the TCR-pMHC complex [54]. Whether or not a peptide-MHCII CAR target would display differences between CD4 and CD8 CAR T cells remains to be studied. A recent study by Dotti and colleagues [55] has examined the role of co-stimulation endodomain in the phosphorylation of Lck and showed that CD19-CAR T cells incorporating CD28z endodomains display a greater degree of activation than those employing 41BBz endodomains. This study also highlights that Lck recruitment to the CAR synapse by CD4 or CD8 co-receptors plays a role in triggering basal CAR-CD3z phosphorylation [55].

Perforin and granzyme mediated programmed cell death by apoptosis (characterised by cell shrinkage, blebbing of the plasma membrane and exposure of phosphatidylserine, followed by fragmentation) is thought to be the major mechanism of T cell cytotoxicity. However recent evidence suggests that T cells, and in particular CAR T cells, utilise other programmed necrotic cell death pathways. Necrotic cell death pathways induce inflammation, which may be critical to avoid when designing CAR T immunotherapies targeting brain tumours. Ensuring immunogenicity of target antigens is one step in the development of effective CAR T therapies; however, it is equally vital to understand the immunobiology of dying cancer cells, as the mode of cell death can influence the immunogenicity of the local tumour microenvironment [56]. A more inflammatory tumour microenvironment that promotes release of pathogen-associated molecular pattern molecules (PAMPs) may favour immunomodulatory responses to tumour associated antigens (TAAs) [57], thus having broader implications for the clinical success of various immunotherapies. Our study presents the first functional study of the CAR: peptide MHC synapse; this analysis of live cell imaging has potential to inform future CAR design and the desired mechanism of cell death with CAR. First, our novel CAR recognising peptide-MHC formed a ‘classical’ ‘bulls-eye’ structure of SMAC proteins at the immune synapse, in contrast with evidence that CAR-protein interactions which display microclusters of Lck [25]. Interestingly, previous studies have also demonstrated that the choice of intracellular co-stimulation domain in the CAR (i.e., CD28 versus 41BB) does not influence immune synapse formation per se [58], but can influence the equilibrium of phosphorylation and dephosphorylation of the CAR-CD3z endodomain and directly influence CAR T cell activation [55]. Previous studies have also shown that varying the length of CARs [59,60] and also the numbers and location of ITAM CD3 [61] can also impact CAR performance.

We have shown in this study that our novel H3.3K27M-HLA-A*02:01-specific GCT615-CAR T cells do mostly induce apoptosis, an ordered, programmed mode of cell death that is thought to result in less inflammation to surrounding cells and tissues [62]. This may confer benefits in translation to the clinic, as the anatomically confined space of the brain and brainstem mandates minimal inflammation with immunotherapy, with the clinically morbid side effects of cerebral oedema and potential brainstem herniation to consider. A smaller proportion of target cells also died via morphological pyroptosis in our study. This is interesting given a recent report from Liu et al. [39] which demonstrated that CAR T induced death via pyroptosis, a mode of cell death typically associated with infection, was mediated by the gasdermin E protein, leading to caspase 1 activation and triggering the inflammatory cascade of cytokine release syndrome [39]. It would be interesting in future to dissect the role of CAR affinity and functional avidity in inducing alternative models of target cell death for future immunotherapy approaches. It is also tempting to speculate that a reduction in affinity of the GCT615-CAR scFv for the H3.3K27M-HLA-A*02:01 complex may enhance the cytotoxic activity of the CAR T cells; such work will be the focus of future study.

In summary, we have demonstrated effective functional targeting of the H3.3K27M-HLA-A*02:01 peptide-MHC complex with a CAR, and that accessory proteins contained within the immune synapse, which influence productive signalling, resemble those mediated by TCR.

## Data Availability

Data analysed in the study is available upon request.

## Supplementary Materials

The following are available online at https://www.mdpi.com/article/10.3390/biomedicines9121875/s1, Figure S1: Scoring of Immune Synapse features; Video S1: GCT615-CD8^+^ CAR T cell killing T2 targets resulting in apoptosis; Video S2: GCT615-CD8^+^ CAR T cell killing T2 targets resulting in necroptosis.
