# Supplementary material for: A Novel Peptide-MHC Targeted Chimeric Antigen Receptor T Cell Forms a T Cell-like Immune Synapse
Source: Biomedicines. 2021 Dec 10;9(12):1875. doi: 10.3390/biomedicines9121875 (PMC8699022; doi:10.3390/biomedicines9121875)
Supplement: Supplementary file 1 [file biomedicines-09-01875-s001.zip › biomedicines-1485707-supplementary/biomedicines-1485707-supplymentary/Figure S1.pdf]

**Supplementary Figure S1.** Scoring of immune synapse features. Criteria on scoring of immune synapse features (score from 0 to 3 points): morphology, *γTubulin polarisation*, *Lck clustering* and *actin depletion*. Higher scores are given based on increasing similarity to physiological T cell immune synapses.

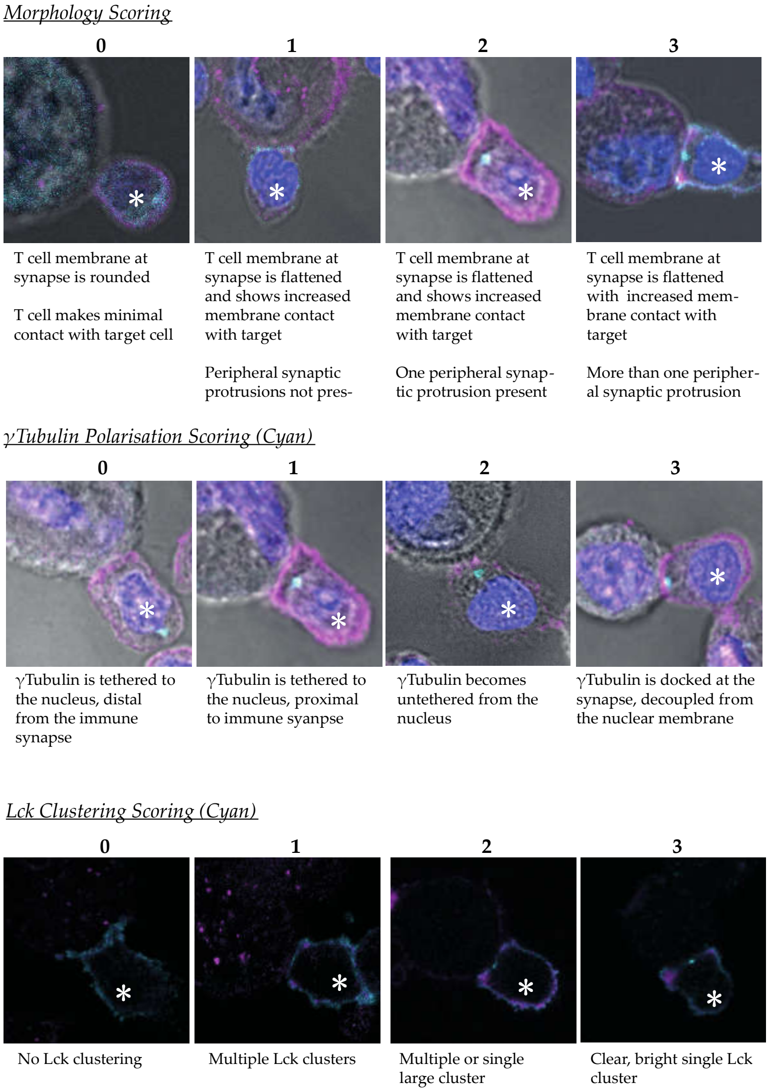

*Actin Depletion Scoring (Magenta)*

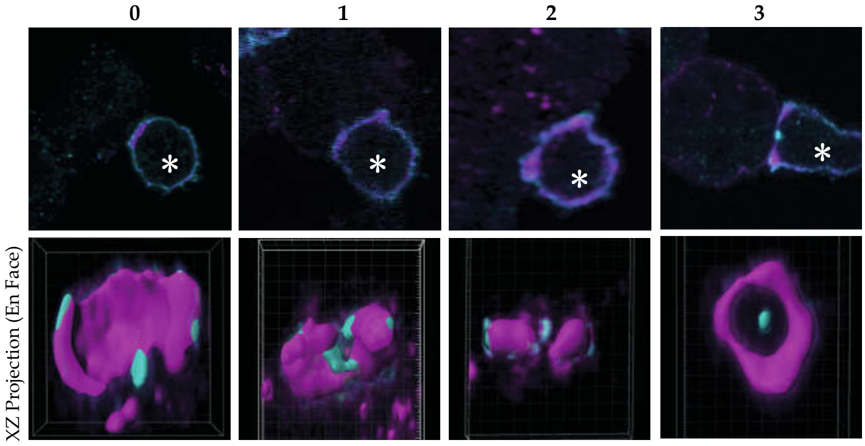


| 0 | 1 | 2 | 3 |
|---|---|---|---|
| Actin acumulates at the synapse | Some actin depleted at the centre of the synapse<br><br>Some accumulation at the edges | Actin depleted at the centre<br><br>Patchy actin accumla-tion at the edges of synapse<br><br>XZ projection of actin ring is incomplete | Actin is depleted at the centre<br><br>Actin accumulates at the edges<br><br>XZ projection of actin ring is complete |
